# Supplementary material for: Neurodevelopment in children born to women exposed to pesticides during pregnancy
Source: PLoS One. 2025 Dec 19;20(12):e0326007. doi: 10.1371/journal.pone.0326007 (PMC12716740; doi:10.1371/journal.pone.0326007)
Supplement: Attachment — Submitted filename: Response matrix 071125.docx [file pone.0326007.s005.docx]

**Response to reviewer’s comments**

We are grateful to the editors and the reviewers for their time and insightful comments on our manuscript titled “*Neurodevelopment in children born to women exposed to pesticides during pregnancy*”. Their feedback has been invaluable in strengthening our manuscript. We have carefully considered all points raised by reviewer 2 and have made revisions in the manuscript in response to the comments. Point-to-point response to the comments is summarized in the table below. The page numbers and lines refer to the manuscript with track changes.

**Table 1: Response matrix to reviewer’s comments**

| **Reviewer 1** | **Author’s response** |
| --- | --- |
| All comments have been addressed. No additional comments | We thank the reviewer after being satisfied with our response to the provided comments and for indicating that they have no further concerns following our revisions. We appreciate for the valuable contribution to improving our manuscript. |
| **Reviewer 2** |  |
| Please specify - LL. 114-115: "and the like" needs further clarification. | We appreciate the reviewer’s suggestion. The vague phrase “and the like” has been replaced with a clearer description of additional complications to enhance precision and readability. The revised sentence on page 5, line 100 reads:  *“Delivery complications included conditions such as obstructed labor, severe bleeding and sepsis, as reported by the mother.”* |
| A minor grammatical error in LL. 258: "evidence" is usually treated as uncountable/singular. | We thank the reviewer for this valuable comment. The grammatical error has been corrected by replacing “there are evidences” with “there is evidence.” This is on page 17, line 244. |
| Also, LL. 67 and 259 - "some of the neurotoxic pesticides are in use" needs to be more specific; readers are entitled to understand which types of pesticides are regarded to as "some of them". Also the Mrema's finding would better be expanded with numerical information either in introduction or discussion. | We thank the reviewer for this valuable comment. The sentence has been revised to specify the types of pesticides and to include quantitative information from reference 16 (Mrema et al). This is added on page 17, lines 244-248. |
